# Physical Biomarkers of Disease Progression: On-Chip Monitoring of Changes in Mechanobiology of Colorectal Cancer Cells

**DOI:** 10.1038/s41598-020-59952-x

**Published:** 2020-02-24

**Authors:** Fern J. Armistead, Julia Gala De Pablo, Hermes Gadêlha, Sally A. Peyman, Stephen D. Evans

**Affiliations:** 1 Molecular and Nanoscale Physics group, Department of Physics and Astronomy, University of Leeds, Leeds, UK; 2 Department of Engineering Mathematics, University of Bristol, Bristol, UK

**Keywords:** Biophysics, Cancer, Cell biology

## Abstract

Disease can induce changes to subcellular components, altering cell phenotype and leading to measurable bulk-material mechanical properties. The mechanical phenotyping of single cells therefore offers many potential diagnostic applications. Cells are viscoelastic and their response to an applied stress is highly dependent on the magnitude and timescale of the actuation. Microfluidics can be used to measure cell deformability over a wide range of flow conditions, operating two distinct flow regimes (shear and inertial) which can expose subtle mechanical properties arising from subcellular components. Here, we investigate the deformability of three colorectal cancer (CRC) cell lines using a range of flow conditions. These cell lines offer a model for CRC metastatic progression; SW480 derived from primary adenocarcinoma, HT29 from a more advanced primary tumor and SW620 from lymph-node metastasis. HL60 (leukemia cells) were also studied as a model circulatory cell, offering a non-epithelial comparison. We demonstrate that microfluidic induced flow deformation can be used to robustly detect mechanical changes associated with CRC progression. We also show that single-cell multivariate analysis, utilising deformation and relaxation dynamics, offers potential to distinguish these different cell types. These results point to the benefit of multiparameter determination for improving detection and accuracy of disease stage diagnosis.

## Introduction

Disease can induce changes to the biological constitutents of cells, such as the cytoskeleton, leading to changes in whole cell-deformability^1^. Thus, cell mechanical phenotyping can be used to study these changes. Distinct mechanical responses have been correlated to a variety of diseases, including; sepsis^2^, malaria^3^, diabetes^4^, sickle cell anaemia^5^ and cancer^6–8^. Specifically, cancer cells have been shown to be softer than normal cells, and deformability increases with metastasic progression^9–11^. A number of techniques have been used for determining cell deformability including; atomic force microscopy (AFM)^6,12^, optical stretching^13^, magnetic twisting cytometry^14,15^, micropipette aspiration^3,16^, and microfluidics^1^.

The different approaches perform deformation over a range of lengthscales (from whole cell to local measurments), timescales and magnitudes of force, subjecting the cells to either tensile, compressive or shear forces. The combination of these factors has resulted in a wide-range of mechanical properties being reported in the literature^12,17^. Additionally, several of these techniques have limited throughput as pre-selection of each single cell is required^6,13,15,16^. The mechanical properties of cells is inherently heterogeneous as their subcellular structure continuously changes through the cell-division cycle, as well as their disease state. To accurately compare deformability between cell types, a high-throughput approach is needed. This requirement lead to the development of microfluidic based methods to hydrodynamically deform cells, displaying high-throughput (n > 1000) and requiring small sample volumes^18–22^. The mechanical response of cells deformed microfluidically is known to depend on the flow regime.

Previous works indicate that different microfluidic flow regimes can lead to a very different mechanical response of cells. Gossett *et al*.^18^ developed Deformability Cytometry (DC)^18,22–25^, in which cells are hydrodynamically stretched at the stagnation point (SP) of an extensional flow device. In these devices, cells undergo deformation due to a compressional force $({F}_{c})$ due to fluid inertia, and a shear force (${F}_{S}$) due to fluid viscosity. By using high-Reynolds numbers (*Re* ≫ 1), ${F}_{C}$ was ~1000 times greater than ${F}_{S}$ resulting in an *inertia-dominant flow regime*^18^. In this regime, increased deformability was found following lymphocyte activation and in stem cell pluripotency, i.e. cell states that are characterised by loose, open, chromatin structures^23^. Treatment of these cells with cytoskeletal altering drugs showed negligible changes to cell deformability thus leading to the conclusion that these flow conditions are primarily sensitive to nuclear structural changes^18,26^. Guillou *et al*. used a similar microfluidic device geometry to deform cells, but in a *shear-dominant flow regime* (*Re* ≪ 1)^27^. Here, treatement with the actin-disrupting drug cytochalasin D showed an increase in cell deformability. Real-Time Deformability Cytometry (RT-DC) utilized a strong velocity gradient across a channel which is only slightly larger than the cell, inducing small bullet-like deformations due to shear-confinement^20,28–31^. RT-DC was able to detect deformability due to cytoskeletal disruption but was insensitive to nuclear changes^29^. Microfluidic approaches that impose shear-force deformation consistently show sensitivity to changes in the cytoskeleton.

**Figure 1:**
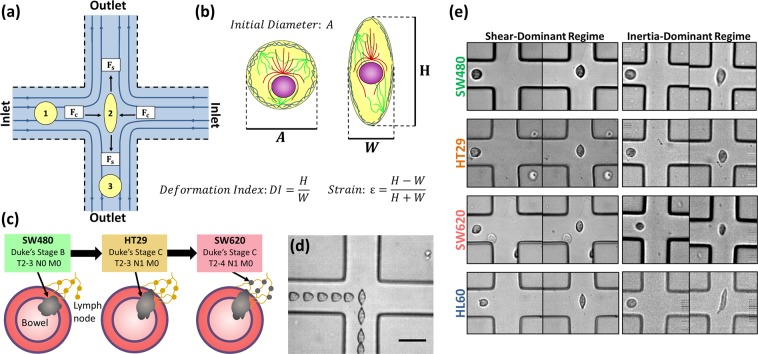
(**a**) Schematic of cross-flow region. (**b**) Schematic of a cell including the nucleus and cytoskeletal filaments (actin, microtubules and intermediate filaments) which are the main contributors of cell stiffness. Also included are parameters extracted from high speed videos of cell deformation: ${A}$ is the initial diameter of the cell before it deforms, $H$ is the height of the cell and *W* is the width of the cell. (**c**) Schematic describing the model for colorectal cancer progression using the three cell lines: SW480, HT29 and SW620. (**d**) Superimposed bright field images showing a single cell entering (from left) the stagnation point (SP) of an extensional flow device and exiting to the bottom of the chamber. (**e**) Example images of deformation events of SW480, HT29, SW620 and HL60 cell lines. Including an image before the cell is deformed and an image of deformation at the SP, for a shear-dominant and inertia-dominant-flow regime. Images from the shear-dominant regime were at a flow rate of Q = 50 µl/min with suspension buffer viscosity of µ = 33 cP, and in the inertia dominant regime Q = 600 µl/min and µ ≈ 1 cP.

We recently demonstrated that by varying flow-rate and cell suspension buffer viscosity, cells can be deformed using both *inertia-dominant* and *shear-dominant* flow regimes using the same microfluidic cross-slot geometry (Fig. 1a)^32^, whereas previous works were limited to either an *inertia-domiannt* or a *shear-dominant* regime^27,33–36^. Cells were deformed in both flow regimes at low and high strains to probe which conditions were most sensitive to cytoskeletal changes. Results showed that a shear-dominant and low-strain regime was most sensitive to cells becoming softer when treated with the actin destabilizing drug latrunculin A, comparatively the inertia-dominant and high strain regime could not detect any changes. We also tracked cell deformation and recovery as a function of time in the shear-dominant regime. This allowed multiple characteristic parameters to be extracted and used to distinguish cell lines, including determination of elastic modulus, plasticity and cell deformation and recovery times.

AFM studies have previously shown a reduction in the elastic modulus of a metastatic CRC cell line (SW620) compared to one deriving from a primary tumour (SW480), showing that phenotyptic softening occurs with metastatic progression due to changes in the actin cytoskeleton^37,38^. Here, we provide the first example of CRC mechanophenotyping using a microfluidic technique. These results offer further insight into metastasis due to being performed across a different range of timescales and forces, as well as being performed on a single cell suspension as opposed to cells adhered to a surface.

Three cell lines (SW480, HT29 and SW620) were chosen as a model for cancer progression (Fig. 1c). The Duke’s staging system is often used to classify CRC stages, however the TNM system is more widely used for all cancer types. TNM classification provides three numbers, where T refers to the primary tumour size, N the number of cancerous lymph nodes, and M the level of metastasis. SW480 derive from a primary adenocarcinoma of Dukes stage B (T2–3 N0 M0 grade), HT29 derive from a more advanced adenocarcinoma of Dukes stage C (T2–3 N1 M0 grade) and SW620 from a lymph node metastasis of Dukes stage C (T2–4 N1 M0 grade)^39^. Figure 1d shows an example deformation event with overlayed images of a cell at various positions in the extensional flow junction, where maximum deformation occurs at the SP. The three CRC cells were deformed in a shear and inertia dominant flow regimes. Images of deformation at the SP are provided in Fig. 1e, highlighting the differences in initial size and deformation between cell lines and regimes. By probing both regimes we show that these cell lines are best differentiated from each other using shear-dominant flow conditions.

By tracking the deformation and relaxation of the cells, multiple characteristic parameters were extracted. The results were also compared to HL60 (human leukemia) cells, used as a representative for a non-epithelial cell type and showed a softer phenotype compared to the CRC cell lines (Fig. 1e). The CRC cell lines showed increased softness with metastatic progression and the elastic modulus determined for each cell type gave values comparable to previous works using AFM^37,38^. These results consolidate the liklelyhood that cytoskeletal changes during development of metastatic phenotypes leads to mechanical changes of the cell. Further, single cell analysis was performed on four datasets associated to SW480, HT29, SW620 and HL60 cells to confirm the statistical significance of the different parameters for distinguishing cell types, indicating that multiple parameters are needed to accurately separate each of these cell types. The single cell analysis points the way for future determinations of inherent cell heterogeneity and its role in response to therapy.

## Results

**Figure 2:**
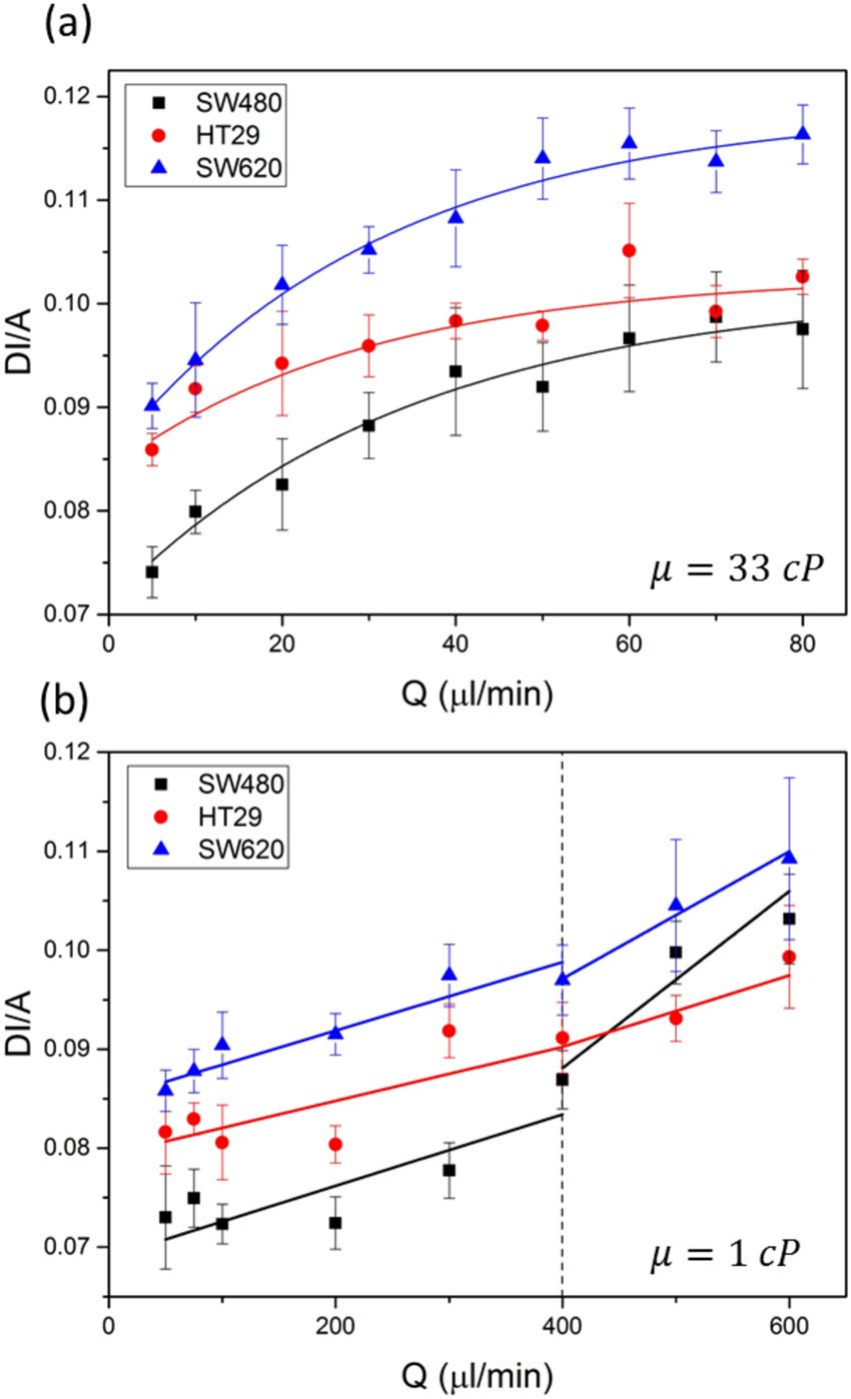
Size normalized deformation index (*DI/A)* for three colorectal cancer cell lines over a range of flow rates (µl/min). $DI/A$ was averaged from datasets from 3 separate experiments combined. (**a**) The flow regime was shear dominant, the viscosity of the cell suspension buffer was 33 cP. The total number of events measured was: *93* < *n* < *931* for SW480, *160* < *n* < *596* for HT29 and *280* < *n* < *734* for SW620. (**b**) The flow regime was inertia dominant, the viscosity of the cell suspension buffer was 1 cP. The total number of events measured was: *30* < *n* < *603* for SW480, *47* < *n* < *619* for HT29 and *30* < *n* < *450* for SW620.

High speed imaging of the three colorectal cancer cell lines was used to capture the maximum deformation index *DI* of the cells at the SP for a range of flow rates, *Q*, in the *inertia* and *shear* dominant deformation regimes. In the *shear* dominant regime the Reynolds number, *Re*, was less than 6 for the entire range of flow rates. In the *inertia* dominant regime, *Re* > *40* for the entire range of flow rates. The deformation index was normalized by the initial diameter of the cells, *A*, to account for variation in cell size^26^. Figure 2a shows *DI/A* as a function of *Q*, in a shear dominant regime where the suspension buffer viscosity was 33 cP. Here, *DI/A* increased asymptotically toward a maximum deformation value ${(DI/A)}_{max}$, which was determined by fitting an exponential function. There is a systematic increase in *DI/A* for SW620 compared to SW480 indicating that under shear dominant conditions the SW620 are significantly more deformable than the SW480 cells. Interestingly, the HT29 display properties similar to those of SW620 at low flow rates, for $Q < 30\,\mu l/min$, but undergo less deformation with increasing flow rate and end up displaying properties close to those of the SW480 for higher flow rates, $Q\ge 40\,\mu l/min$.

Figure 2b shows *DI/A* as a function of *Q*, in a inertia-dominant regime where the suspension buffer viscosity was 1 cP. An abrupt change in behavior can be seen at $Q=400\,\mu l/min$, which was identified as the *yield stress* of the cells and is associated to cytoskeletal breakdown^32^. For each cell line there is a linear relationship between *DI/A* and *Q* for $Q < 300\,\mu l/min$. For flow rates $Q\ge 400\,\mu l/min$ there is also a linear trend, however the gradient increases for both SW480 and SW620. For $Q < 400\,\mu l/min$, SW620 are the most deformable and SW480 are the least deformable. However, for $Q\ge 400\,\mu l/min$ the cell lines are less distinguishable from each other. Figure S1 shows the unnormalized data, *DI* as a function of Q, for the three cell lines in both shear and inertia-dominant regimes.

**Figure 3:**
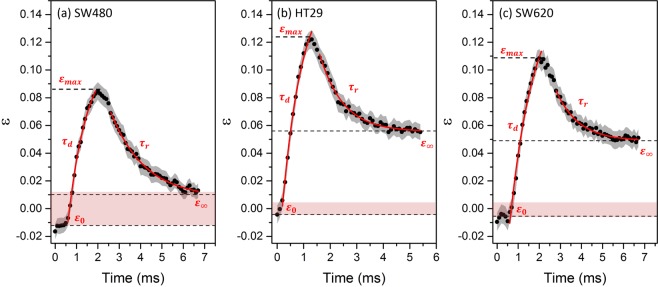
Strain *ε* was tracked for the three CRC cell types as a function of time, with the standard error shown by the grey shaded areas. Q was fixed at 5 µl/min and the suspension medium viscosity was 33 cP. The final strain is marked by dashed lines $({\varepsilon }_{\infty })$, found by extrapolation of a exponential fit to the relaxation (red line). (**a**) The averaged deformation trace of N = 56 SW480 cells. (**b**) The averaged deformation trace of N = 49 HT29 cells. (**c**) The averaged deformation trace of N = 50 SW620 cells.

Averaged deformation traces were measured for each of the 3 cell lines, supplementary videos 1–3 show example deformation events. Figure 3 shows the average strain $\varepsilon$ as a function of time for SW480, HT29 and SW620 cells as they deform and recover in the extensional flow device. Cells were deformed in a shear-dominant and low velocity regime ($\mu =33.4\pm 0.3\,cP\,{\rm{and}}\,Q=5\,\mu l/{\rm{\min }}$). Multiple parameters were determined from the averaged deformation traces of N = 56 SW480, N = 49 HT29 and N = 50 SW620, which are labelled in the traces in Fig. 3 and values are summarised in Table 1. *A* is the initial cell diameter, ${\varepsilon }_{max}$ in the maximum strain, ${\varepsilon }_{0}$is the initial strain before entering the SP and ${\tau }_{d}$ is the deformation time as the cell deforms. After cells reached their maximum strain ${\varepsilon }_{max}$ near to the SP, the strain began to decrease exponentially as the cells traversed the outlet channel. A cell relaxation time, ${\tau }_{r}$, was extracted from an exponential fit, as well as a final strain ${\varepsilon }_{\infty }$ from extrapolation of the fit. The strain values are vectors due to the inlet channels being perpendicular to the outlet channels, hence some ${\varepsilon }_{0}$ values are slightly below 0 due to small deformations induced by shear channel confinement. Thus, if cells recover their original shape after deformation the magnitude of initial strain should be the same as the magnitude of final strain ($|{\varepsilon }_{0}|=|{\varepsilon }_{\infty }|$). The areas shaded in pink in Fig. 3 are a width of $2|{\varepsilon }_{0}|$, and denote whether cells recover their initial strain.

**Table 1:** Multiple characteristic parameters for HL60 (N = 50), SW480 (N = 56), HT29 (N = 49) and SW620 (N = 50) cell lines, extracted from the deformation traces of cells deforming at the stagnation point of an extensional flow at 5 µl/min in the shear regime (µ ≈ 33 cP).

	HL60	SW480	HT29	SW620
${\varepsilon }_{max}$	0.18 ± 0.01	0.08 ± 0.01	0.12 ± 0.01	0.11 ± 0.01
${\tau }_{r}({\rm{ms}})$	3.52 ± 0.14	1.36 ± 0.06	0.89 ± 0.05	1.04 ± 0.05
${\tau }_{d}\,({\rm{ms}})$	1.04 ± 0.05	0.89 ± 0.10	0.76 ± 0.10	1.15 ± 0.20
$E\,(Pa)$	301 ± 29	542 ± 66	309 ± 50	372 ± 98
${\varepsilon }_{0}$	−0.012 ± 0.004	−0.012 ± 0.006	−0.004 ± 0.007	−0.007 ± 0.0071
${\varepsilon }_{\infty }$	+ 0.03 ± 0.009	+ 0.010 ± 0.003	+ 0.056 ± 0.001	+ 0.049 ± 0.001

The elastic modulus *E* was extracted using the Kelvin-Voigt model (Eq. 4). Figure S2 shows the velocity profiles calculated along the central axis of the extensional flow junction, that were fitted with a *sine* function and used to fit the Kelvin-Voigt model to the deformation trace (shown in red in Figure S2). As a comparison, control data from the averaged deformation trace of N = 50 HL60 cells is also included in Table 1 from Armistead *et al*. (2019)^32^.

**Figure 4:**
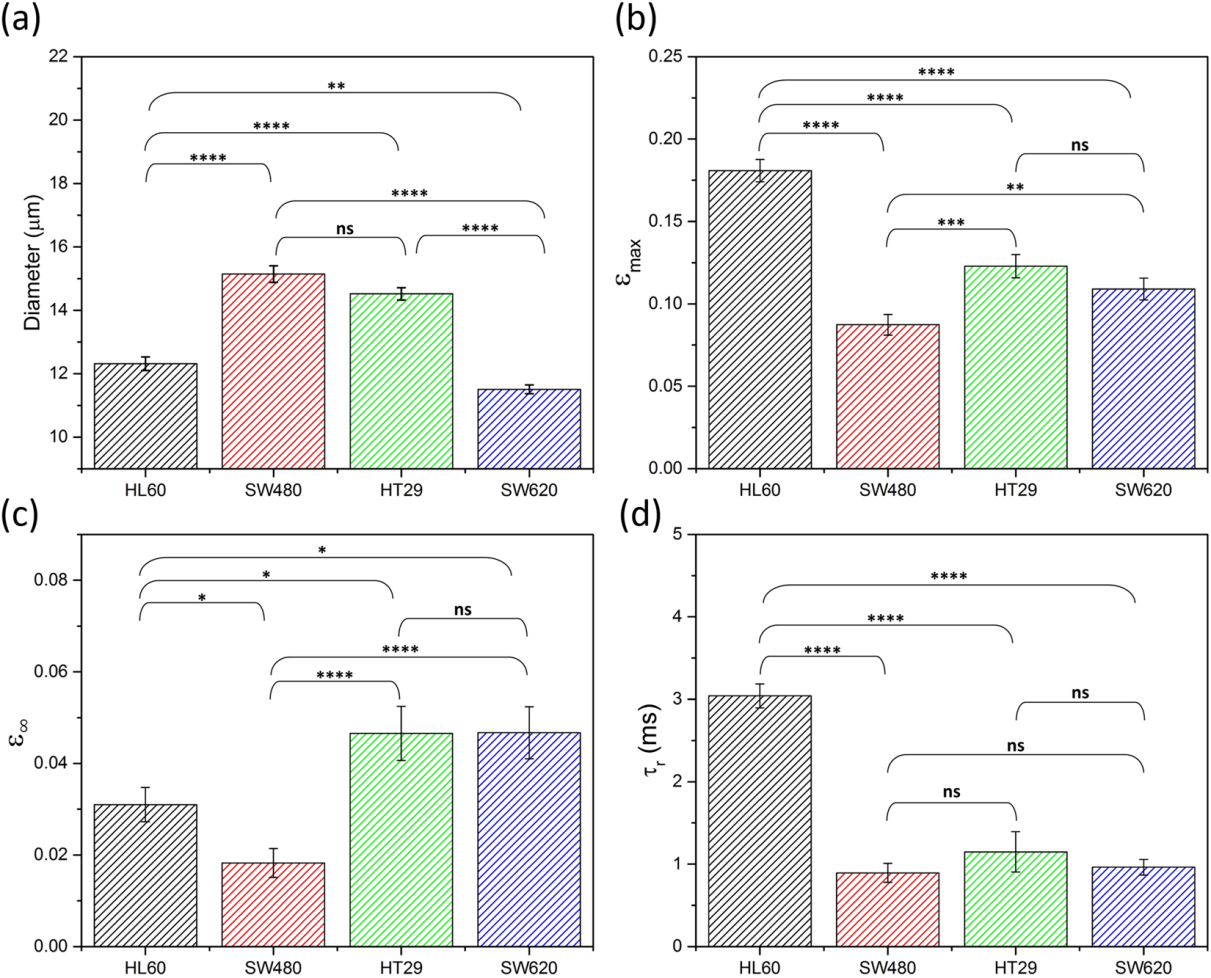
Multiparameter analysis of HL60, SW480, SW620 and HT29 cell populations. The error bars denote the standard error SE, statistical t-tests were done to determine the level of significance. (**a**) Initial cell diameter, (**b**) The maximum strain ${\varepsilon }_{max}$, (**c**) the final strain ${\varepsilon }_{\infty }$ and (**d**) the relaxation time ${\tau }_{r}$ were extracted from deformation traces of single cells deforming at 5 µl/min in a shear dominant regime (~33 cP).

Single cell analysis (SCA) was undertaken on the datasets extracted from tracking $\varepsilon$ as a function of time for each of the cell lines. Two sample t-tests were performed on 5 of the parameters to quantify the statistical significance between the different cell lines when using these parameters^40^. Figure 4 shows a bar graph of the average *A*, ${\varepsilon }_{max}$, ${\varepsilon }_{\infty }$, ${\tau }_{r}$ of the 4 cell lines and indicates the level of significance: where p > 0.05 is not significant (ns), 0.01 < p < 0.05 is significant (*), 0.001 < p < 0.01 is very significant (**), 0.0001 < p < 0.001 is extremely significant (***) and p < 0.0001 is extremely significant (****), raw values can be found in Table S1. The data for ${\varepsilon }_{0}$ is shown in Figure S6.

**Figure 5:**
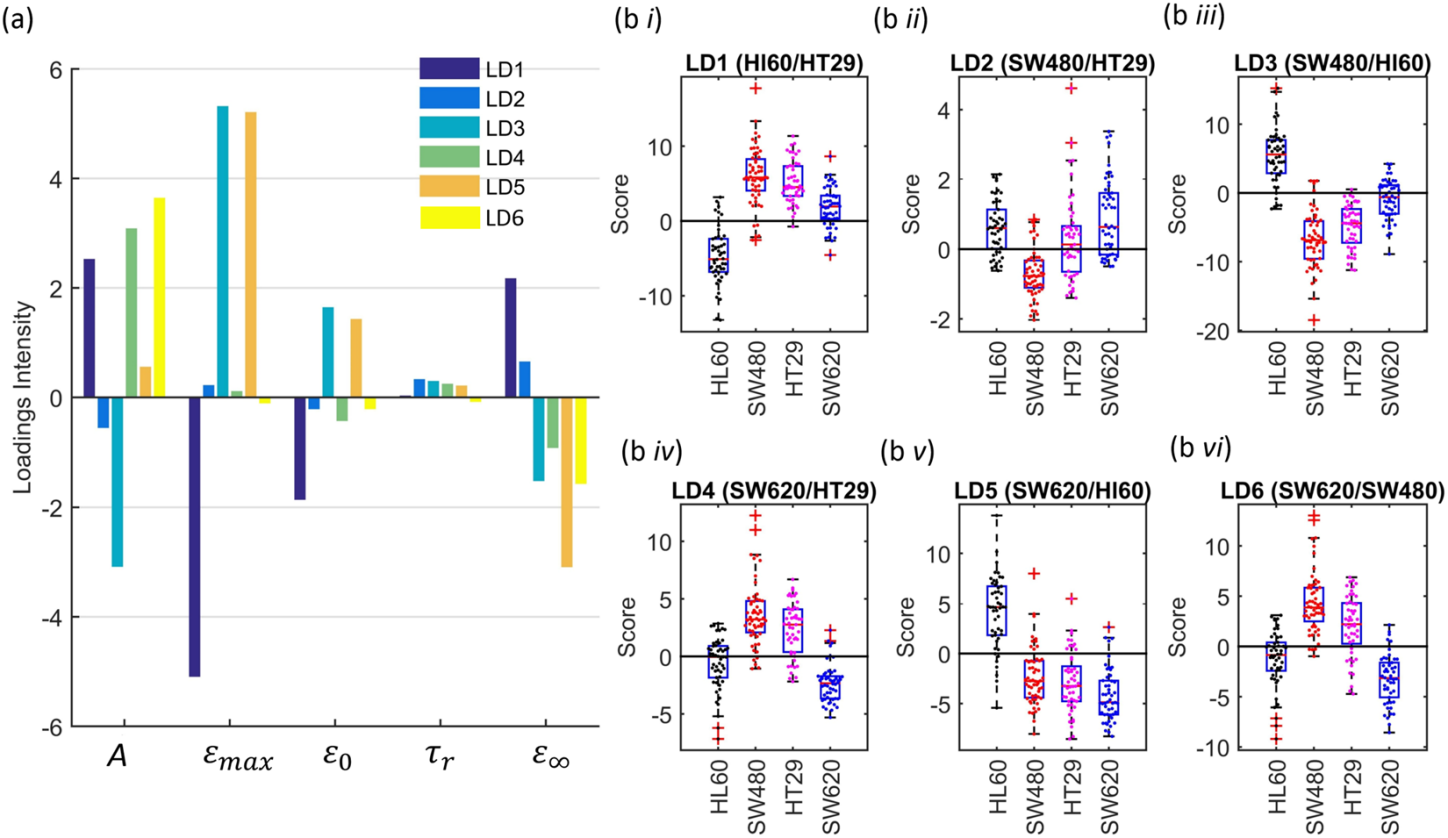
Linear discriminant analysis of the four cell lines; HL60, SW480, HT29 and SW620. Where the bar plot indicates the loadings for each of the linear discriminats (LD) (**a**), and the box plots and beeswarm plots on the right correspond to the scores on each of the LDs (**b** i-vi).

Linear Discriminant analysis (LDA) was also used on the four datasets to test the abilities of these parameters for classifying the different cell types (Fig. 5). LDA is a supervised multivariate method that obtains a linear combination of the parameters that best separates the different cell lines. When trained on a 4-class dataset (4 cell types), where each linear discriminant (LD) maximizes the separation of a pair of classes, using all the LDs scores for the final classification. A k-fold validation test was applied to the data, where a random fraction of the data was used to train the LDA model. This model was then applied to the remaining data to test the models ability to correctly classify the cell types based on the given parameters. Here, a 5-fold validation test was applied, where the loadings and scores of the LDs are shown in Fig. 5 and the confusion matrix is shown in Table 2. LD1, LD3 and LD5 showed the best separation between HL60 and the three CRC cell lines, with individual scores shown in Figure 5bi, iii and v. These discriminants were all marked by a higher maximum strain ${\varepsilon }_{max}$ of the non-adherent cells compared to the adherent cells, giving good classification of HL60 compared to the CRC cell lines. The final strain ${\varepsilon }_{\infty }$ discriminated HT29 and SW620 from HL60, and initial diameter showed good separation of HL60 from HT29 and SW480.

**Table 2:** k-fold validation tests to classify the four cell lines (5-fold). Shows that 100% of the HL60 can be classified correctly as HL60, and that 85% of SW480 were classified correctly. HT29 and SW620 were harder to classify, the average classification of the four cell lines was 69 ± 1%. The rows represent the actual cell type and the columns represent the predicted cell type.

	HL60	SW480	HT29	SW620
HL60	82 ± 2	2 ± 1	5 ± 1	10 ± 2
SW480	9 ± 1	71 ± 3	15 ± 2	5 ± 1
HT29	5 ± 2	39 ± 3	36 ± 3	20 ± 3
SW620	8 ± 2	2 ± 1	6 ± 1	85 ± 2

Between the CRC cell lines, LD2 and LD6 showed the best separation between SW480 cells with HT29 and SW620 cells respectively. LD2 showed higher relaxation time ${\tau }_{r}\,\,$of SW620 and HT29 cells when compared to SW480 cells, accompanied by higher final strain ${\varepsilon }_{\infty }$ and maximum strain ${\varepsilon }_{max}$ but lower initial strain ${\varepsilon }_{0}$ and diameter *A*. LD6 and LD4, comparing SW620 cells with either SW480 or HT29 cells, mainly classified according to diameter *A*, showing that SW620 cells were smaller. When using 5-fold LDA classification, 82% of the HL60, 71% of SW480 and 85% of SW620 cells were correctly classified by the model. HT29 cells were more difficult to accurately classify, 36% correctly classified with 39% incorrectly classified at SW480 and 20% as SW620. Of the 4-class dataset, the average classification rate was ~69%.

## Discussion

Three colorectal cancer cell lines representing different stages of disease progression from Dukes primary stage B (SW480) through to metastasis to the local lymphatic system (SW620) Dukes stage C were studied in the *inertial* and *shear* regimes. HT29s represent an intermediate state of Dukes stage C, primary tumour. In the *inertial* regime, (Fig. 2b) for *Q* < 300 *μl*/*min* the size normalized deformation, *DI/A*, was greatest for the SW620 and least for the SW480. For *Q* > 300 *μl*/*min*, the cell lines become indistinguishable by *DI/A* suggesting that *Q* ≅ 400 *μl*/*min* represents the cell *yield stress*. In Armistead *et al*. (2019) we showed that cell softening due to disruption of the cytoskeleton using LatA was only measurable at flow rates below the *yield stress* of HL60 cells. This corroborates that cytoskeletal changes are associated with CRC progression, and cytoskeletal breakdown above the *yield stress* results in no measurable changes to deformability for the higher flow rates.

In the shear regime the SW620 cells were the most deformable for all values of *Q* and the SW480’s the least (Fig. 2a). The HT29 cells tended to be similar to those of the SW620s at low flow rates but their *DI/A* values approached those of the less deformable SW480’s for higher flow rates, 𝑄 ≥ 40 *μl*/*min*. The secondary tumor cells being softer than the primary cells is a result seen in previous works^37,38^. Several papers report that metastatic SW620 cells up-regulate genes associated with cytoskeletal changes, particularly related to the actin, which accompany increased motility, enhanced invasive potential, higher proliferation and reduced adhesion compared to the non-metastatic SW480^41–45^.

We note, that had we characterized the deformation without adjusting for cell size, these cells would not be distinguished from each other based on their *DI* index alone in either the inertial or shear regimes (Figure S1). In the inertial regime, the SW480 showed increased *DI* compared to HT29 and SW620 for Q > 400 µl/min. However, the SW480 are the largest cell (Table 1) and this would make them appear more deformable above the yield stress associated with subcellular disruption of actin filamnets (<400 µl/min). Figure S6 shows the average *DI* of these cell lines when deformed at 5 µl/min in the shear regime. Here, SW480 had the smallest *DI* and HT29 the largest *DI*. A two sample t-test was used to quantify whether the differences in *DI* were significant, this showed that the p-value between the *DI* of HT29 to the other cell lines was extremely significant (***). Whereas the p-value between SW480 and SW620 was > 0.05 and was not significant.

We have previously shown that a low-strain and shear dominant regime is most sensitive to cytoskeletal changes^32^. Thus, to study mechanical changes in our CRC model system we performed deformation tracking and multiparameter analysis of SW480, HT29 and SW620 deforming at Q = 5 µl/min, in the shear regime. The general trait of SW620 being softer and more deformable than SW480 cells was also evident in the parameters determined from the deformation traces (Table 1) with the SW620 having a lower mean elastic modulus than SW480’s, and a higher maximum strain *ε*_*max*_. Palmieri *et al*.^37^ noted that SW480 cells have two appearances in culture, an epithelial-type morphology (E-type) and a rounded morphology^37^. Using AFM they found the elastic modulus of SW480 E-type to be 1060 Pa and SW480 R-type to be 580 Pa. The elastic modulus determined by AFM for SW480 R-type cells was within error the same as that determined here using microfluidics. Further, both AFM and microfluidics show the metastatic cells to be softer than the non-metastatic (SW480). Additionally, the elastic modulus determined for the SW620 cells (Table 1) is within error of the value reported using AFM^37^. Tsikritis *et al*. 2015 also measured the elastic modulus of SW480 and SW620 using AFM, finding SW620 to be ~3 fold softer^38^.

SW480 and HT29 are closest in terms of initial diameter compared to SW620, however *ε*_*max*_ and the elastic modulus of HT29 and SW620 are comparable whereas SW480 is significantly higher. These results suggest that as colorectal cancer progresses from Duke stage B to C (SW480 to HT29), requiring the cells to migrate toward the outer lining of the bowel, the cells undergo structural changes and become softer. The similarity between HT29 and SW620 is a possible indicator that as the cells then move from the outer lining to a secondary tumour site in the lymph nodes, changes to the cell structure are less essential. Further, the SW480’s also recovered their original strain values after deformation, whereas SW620 and HT29 both recovered to a final strain of ${\varepsilon }_{\infty } > 0.04$ which is significantly higher than their initial strain ~0. This could indicate an additional slower relaxation process occurring over a longer timescale than our experiments measure, or that the cells have an associated “plastic” deformation. Cell deformation is commonly thought to be a viscoelastic response, however permanent plastic deformations have been seen due to breakdown of the cytoskeletal scaffold^46^. The short relaxation time for the SW620 and HT29 might reflect a more active cytoskeleton. For comparison we note that the HL60 cells, also fully recover to their strain free values.

Single cell analysis was performed on the datasets acquired for obtaining the deformation traces of N = 50 HL60 cells, N = 56 SW480 cells, N = 49 HT29 cells and N = 50 SW620 cells. The significance of the parameters *A*, ${\varepsilon }_{max}$, ${\varepsilon }_{\infty }$, ${\tau }_{r}$ and ${\varepsilon }_{0}$ were tested using two sample t-tests to obtain p-values, shown in Table S2 and summarised by Fig. 4 and Figure S7. We first note that the initial strain ${\varepsilon }_{0}$ of the four cell lines are within error and show no significant difference (Figure S7), additionally the deformation time ${\tau }_{d}$ showed no change between cell lines and thus was not studied using SCA. Statistical significance is shown in separating all the cell lines from each other using their initial diameter *A* (Fig. 4a) and their maximum strain (Fig. 4b) These results show that even though SW480 and HT29 cannot be separated by their initial size, they can be identified by their maximum strain. It also corroborates previous discussions that SW620 and HT29 have similar deformability. Fitting the cell recovery with an exponential function was used to extrapolate values of final strain ${\varepsilon }_{\infty }$ (Fig. 4c) and their relaxation time (Fig. 4d). The final strain ${\varepsilon }_{\infty }$ between SW480 and the other CRC cell lines is extremely significant (p < 0.0001) whereas HT29 and SW620 have no significant difference in ${\varepsilon }_{\infty }$. These results continue to show the trend that SW480 are mechanically different to the later stage CRC cell lines HT29 and SW620, which show indistinguishable deformation and relaxation parameters.

Results show that ${\tau }_{r}$ cannot be used to distinguish between any of the CRC cell lines (all have p > 0.05), but all three CRC cell lines compared to HL60 are extremely significant. HL60 are the most deformable and have a relaxation time ~3 fold larger than the CRC cell lines. The deformation and relaxation parameters (${\varepsilon }_{max},{\varepsilon }_{\infty }$) were able to distinguish SW480 from SW620 and HT20, and the data suggests SW480 is stiffer than the later stage CRC cell lines. Mechanical changes in CRC cell lines has been attributed to changes in the actin. This suggests that ${\tau }_{r}$ may be more sensitive to the mechanics of the nucleus as opposed to the cytoskeleton. Table S3 shows the nuclear diameter and nuclear ratio *(A*_*nucleus*_*/A*_*cell*_) of the four cell lines. The nucleus is known to be mechanically stiffer compared to the rest of the cell^47,48^, and HL60 have a smaller nucleus and nuclear diameter compared to the CRC cell lines. However, the responses are likely more complex due to coupling between ${\tau }_{r}$ and ${\varepsilon }_{\infty }$. As the extrapolated value ${\varepsilon }_{\infty }$ did not recover to the initial value (${\varepsilon }_{\infty }\ne |{\varepsilon }_{0}|$), this suggests longer relaxation processes are at play which could offer more insight into the mechanical response of cells to an applied stress. Overrall, the statistical testing shows that whilst no single parameter can significantly distinguish all cell lines from each other, multiparameter analysis can lead to more classification.

Finally, a 5-fold validation test using LDA showed that HL60, SW480 and SW620 had moderately good classification rates (<71%) However, only 36% HT29 were classified correctly with the majority incorrectly classified as SW480 (39%) or SW620 (20%). HT29 are the intermediate step of the CRC model and were generally harder to distinguish, this may be indicative of these cells having intermediate properties between the two. For instance, results showed that HT29 have comparable initial size to SW480 but deformation and relaxation parameters comparable to SW620 (Fig. 4). The relatively low sample size is also likely to hinder classification rates (n < 56). Overrall, the average correct classification rate of the four cell types was 69% which highlights the need for larger datasets combined with multiparameter analysis for accurate classification of different cell types.

## Conclusion

Three CRC cell lines, representing different states of disease progression, were studied in the shear and inertia-dominant flow regimes. In the shear dominant regime, we measured deformation traces and determined multiple characteristic parameters, including: maximum strain *ε*_*max*_, elastic modulus *E* and relaxation time *τ*_*r*_. Interestingly, the elastic modulus values of each cell line were of the same order of magnitude as previous AFM measurements, in spite of the different modes and timescales of operation. These results showed a general increase in deformability with CRC progression using *ε*_*max*_ and elastic modulus. The cell types could only be differentiated under certain flow conditions, indicating that in the inertial regime only low-strains (below the *yield stress)* are sensitive to cytoskeletal changes whereas high-strains (above the *yield* stress) are not. Additionally, at a critical flow rate in the inertia-dominant regime we saw an increase in gradient of *DI* as a function of flow rate, this occurs due to the breakdown of the cells internal structure, and as such the mechanical properties probed beyond this point would depend only on the viscous properties of the cytoplasm. This corrobated previous works that show that changes to actin structure associated with metastatic progression result in the cells becoming softer.

Our results are the first example of using microfluidic deformation to distinguish between non-metastatic and metastatic CRCs and support the expectation that metastatic cells are more deformable than non-metastatic cells due to cytoskeletal changes. Further, we found that multiple parameters were needed to be able to distinguish the four cell types from each other. We observed that SW620 and HT29 are more deformable and softer than SW480 and do not recover their original strain suggesting that they undergo an additional slower relaxation process, occurring over a time period too long to be captured in our experiments. Single cell and multiple parameter analysis showed changes in the mechanical properties of CRC cells attributed to specific sub-structural changes, and that these can also be used to classify different cell types. Results show that a single-cell high-throughput technique combined with multiparameter analysis will allow us to advance our understanding of cancer progression, and to accurately classify heterogeneous samples of disease states.

## Materials and Methods

### Microfluidic devices

Microdevices were fabricated using standard rapid prototyping and soft lithography techniques, using a Silcon master as a mold for fabricating microfluidic devices in polydimethylsiloxane (PDMS). Piranha wet etch (H_2_SO_4_ & H_2_O_2_) was used to clean a 3-inch Silicon wafer, which was then rinsed with deionized water. A 25 µm layer of photoresist SU-8 2025 (Microchem, Warickshire, UK) was coated onto the wafer. Channels were etched into the SU-8 using direct-write laser lithography (MicroWriter ML™, Durham Magneto Optics), using a 375 nm wavelength laser.

PDMS base and cross-linking agent (SYLGARD 184) were mixed at a 1:10 ratio and poured onto the Silicon master, resulting in a negative replica of the SU-8 structures. This was cured for 1 hr at 75 ^o^C, forming a hydrophobic elastomer which was peeled away from the master. Device inlet and outlet holes were punched using a Biopsy Puncher, then the PDMS was sealed to a glass slide by treatment with Oxygen plasma. Microdevices had a channel height of 25 µm and inlet and outlet channels at the extensional flow junction (Fig. 1a) had a width of 35 µm.

### Characterising flow regime

Cells were deformed in a microfluidic device with a cross-slot geometry, cells passed through the stagnation point SP of an extensional-flow junction where their subsequent maximum deformation was measured using the deformation index DI = H/W (Fig. 1b). A compressive force ${F}_{C}$ and a shear force ${F}_{S}$ act on the cell, where the total force on the cell at the SP can be defined as ${F}_{T}={F}_{S}+{F}_{C}$. Equation (1) was used to determine ${F}_{C}$, where $\rho$ is the density of the suspension media, *U* is the fluid velocity, ${A}_{p}$ is the cross sectional area of the cell and ${C}_{D}$ is the drag coefficient^49,50^. Equation (2) was used to determine ${F}_{S}$, where where $\mu$ is the viscosity of the suspension media, *r* is the cell radius and $\dot{\gamma }$ is the strain rate^18,27^. By tailoring the suspension medium viscosity and the flow rate, the flow regime can be either shear or inertia dominant. This can be described using the Reynolds number *Re*, where *Re* > *40* represents the change from a shear to an inertial regime^22,42,43^. A more detailed description and calculations of the flow regimes can be found in ref. ^32^.

1 $${F}_{C}\cong \frac{1}{2}\rho {U}^{2}{C}_{D}{A}_{p}$$

2 $${F}_{S}\cong \dot{\gamma }\mu (4\pi {r}^{2})=2\pi U\mu r$$

### Experimental procedure

To capture cell deformation on-chip, microfluidic devices were positioned on an inverted bright-field microscope (Eclipse Ti-U, Nikon, Japan), using a 10x objective (with an additional 1.5x magnification for flow rates $Q < 100\,\mu l/min$). High speed microscopy (Photron, Tokyo, Japan) was used at frame rates of 7500–260,000 fps and exposure times of 0.37–6.67 µs. To capture images at high frame rates and low exposure times, an additional light source was mounted above the set-up. Off-line automated image analysis was performed using ImageJ and Matlab, which tracked the shape and position of each cell as a function of time. Utilising a mathematical image processing algorithm adapted from flagellar image tracking^51^. This allowed parameters such as initial diameter, velocity of deformation and other cell shape parameters (Fig. 1b) to be extracted, including the time evolution of the shape dynamics.

Cell events were selected which underwent the same applied stress whilst passing through the extensional flow junction, allowing accurate comparison of deformability between samples. This was achieved by looking at the velocity profile of each cell traversing the extensional flow junction. Exclusion of events was based on the change in velocity, $\Delta v$, (Eq. 3) where ${v}_{inlet}$ is cell velocity in the inlet channel, and ${v}_{min}$ is the minimum cell velocity in the cross flow junction. If a cell perfectly deforms whilst trapped at the SP ${v}_{min}=0$ and $\Delta v=1$. Cells which do not decelerate at all $({v}_{min}={v}_{inlet})$ have $\Delta v=0$. Discarding events with $\Delta v < 0.75$ was chosen as a condition for characterising *DI* of a sample^32^.

3 $$\Delta v=\frac{{v}_{inlet}-{v}_{min}}{{v}_{inlet}}$$

### Calculation of cell elastic modulus

The Kelvin-Voigt model, comprising of a linear spring and a viscous dashpot in parallel, was fitted to the strain as a function of time to determine the elastic modulus of the four cell types. The velocity profile of the cell as it passes the extensional flow junction varies approximately as a sine function, for a period *T*, where $\omega =\frac{2\pi }{T}$ is the angular frequency. In the shear-dominant regime, the deformation velocity is proportional to applied stress $\sigma (t)$ (Eq. 1) and thus the stress also varies as a sine function. This leads to the adapted trigonometric analytical solution of the Kelvin Voigt model, Eq. 4. Where $\varepsilon (t)$ is the strain, *E* is the elastic modulus associated with the linear spring, $\eta$ is the viscosity associated with the dashpot, ${\sigma }_{0}$ is the maximum stress on the cell at the SP^32^.

4 $$\varepsilon (t)=\frac{{\sigma }_{0}}{({\eta }^{2}{\omega }^{2}+{E}^{2})E}(({\eta }^{2}{\omega }^{2}-E\eta \omega +{E}^{2}){e}^{-\frac{Et}{\eta }}-E\eta \omega \,\cos (\omega t)+{\omega }^{2}{\eta }^{2}+{E}^{2}\,\sin (\omega t)+{E}^{2})$$

### Cell culture

The SW480, SW620 and HT29 cell lines were provided by St James’s University Hospital, and had been STR (short tandem repeat) profiled to authenticate cell identity. Cells were cultured in Dulbecco’s Modified Eagle Medium (DMEM F-12, Glibco) supplemented with 10% Fetal Bovine Serum (Sigma), 2 mM Glutamax (Thermo Fisher Scientific) and Penicillin 100 units/ml Streptomycin 100 µg/ml (Sigma). Passage numbers were below 50 for all experiments. All three are adherent cell lines and were detached by incubating in TrypLE (Thermo Fisher Scientific) for 5 mins. They were then centrifuged at 100 g for 4 mins and then resuspended in DMEM or PBS with 0.5% (w/v) methyl cellulose (Sigma, UK).

The HL60 cell line was purchased as a frozen stock (ECACC European Collection of Authenticated Cell Cultures, 98070106) and cultured in RPMI (Roswell Park Memorial Institute) growth media supplemented with 10% Fetal Bovine Serum (Sigma), 2 mM Glutamax (Thermo Fisher Scientific) and Penicillin 100 units/mL Streptomycin 100 µg/mL (Sigma). HL60 are a non-adherent cell line, centrifuging at 100 g for 4 mins was sufficient to visibly pellet the cells which were then gently resuspended in the desired suspension medium. Cells were either suspended in RPMI or re-suspended in PBS with or 0.50% (w/v) methyl cellulose (Sigma, UK) to increase viscosity. The viscosity of the cell suspension buffer (PBS with 0.5% methyl cellulose) was measured using a Rheometrics SR-500 Dynamic Stress Rheometer in the parallel plate configuration (diameter of 25 mm).

## Supplementary information

Supplementary information.

Supplementary Video1.

Supplementary Video2.

Supplementary Video3.
