# Supplementary material for: Physical Biomarkers of Disease Progression: On-Chip Monitoring of Changes in Mechanobiology of Colorectal Cancer Cells
Source: Sci Rep. 2020 Feb 24;10:3254. doi: 10.1038/s41598-020-59952-x (PMC7039955; doi:10.1038/s41598-020-59952-x)
Supplement: Supplementary file 1 — Supplementary information. [file 41598_2020_59952_MOESM1_ESM.pdf]

Physical biomarkers of disease progression: on-chip monitoring of changes in mechanobiology of colorectal cancer cells

Fern J. Armistead,[1] Julia Gala De Pablo,[1] Hermes Gadêlha,[2] Sally A. Peyman,[1] and Stephen D. Evans[1]
[1] Molecular and Nanoscale Physics group, Department of Physics and Astronomy, University of Leeds, Leeds, UK; [2] Department of Engineering Mathematics, University of Bristol, Bristol, UK.

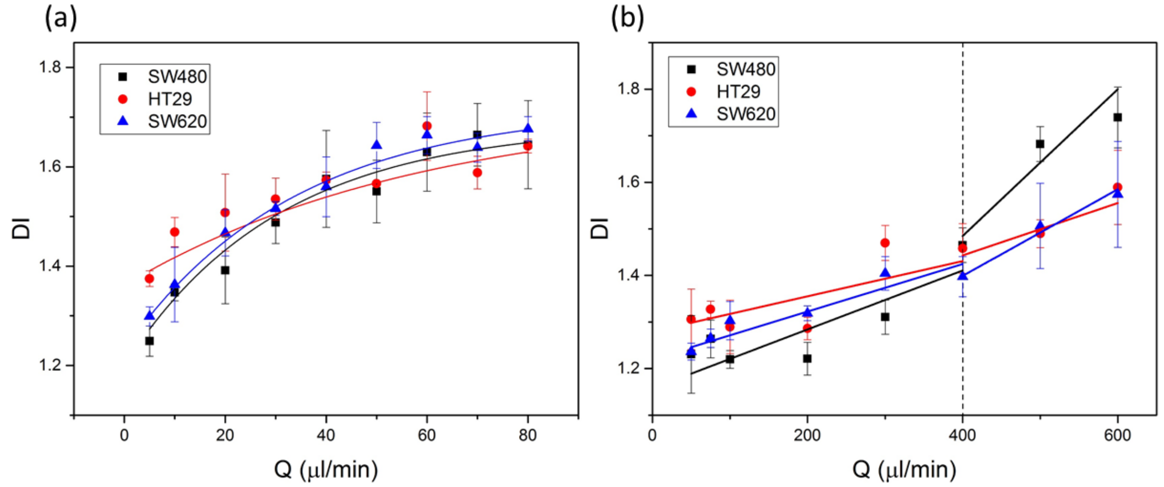

Figure S1: DI of three colorectal cancer cell lines over a range of Q (μl/min): (a) The flow regime was shear dominant, the viscosity of the cell suspension buffer was 33 cP. (b) The flow regime was inertia dominant, the viscosity of the cell suspension buffer was 1 cP. $DI/A \pm SE$ *was averaged from multiple cell events combined from N=3 repeats.*

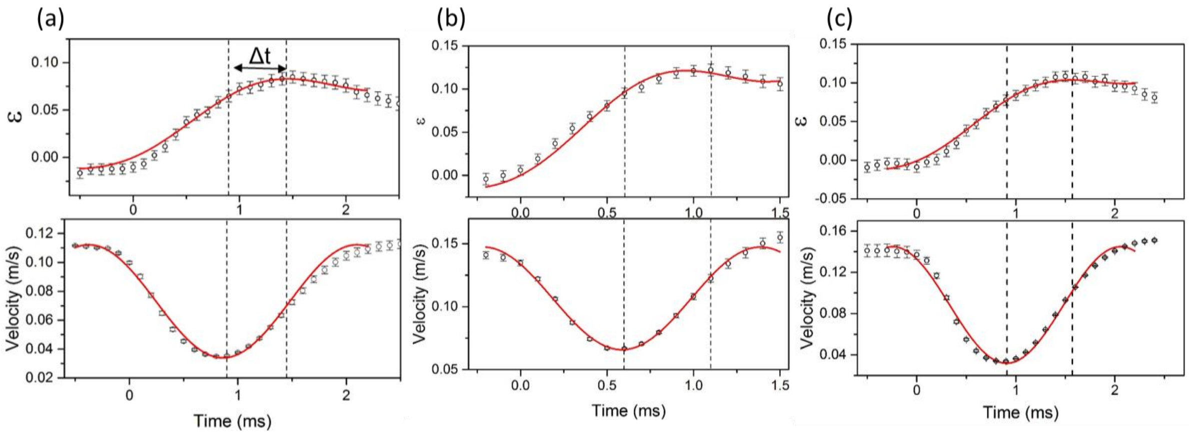

Figure S2: *(a) The strain profile of N=56 SW480 cells, the Kelvin-Voigt model was fitted, shown in red. The average velocity profile of the same 56 cells is shown. A sine function is fitted, shown in red. (b) Strain and velocity profiles for N=49 HT29 cells. (c) Strain and velocity profiles for N=50 SW620 cells. For all datasets Q was 5 μl/min and viscosity was (μ=33 cP).*

Table S1: *Characteristic parameters of HL60, SW480, HT29 and SW620 cell lines, found from the averages of individual deformation traces (single cell analysis SCA) of cells deforming at the stagnation point of an extensional flow at 5 µl/min in the shear regime (µ≈33 cP).*

	HL60	SW480	HT29	SW620
A (µm)	12.3±0.21	15.2±0.25	14.5±0.20	11.5±0.14
ε_{max}	0.18±0.01	0.09±0.01	0.12±0.01	0.11±0.01
τ_r (ms)	3.04±0.15	0.89±0.14	1.15±0.24	0.96±0.10
ε_0	-0.012±0.004	-0.012±0.006	-0.005±0.006	-0.005±0.006
ε_∞	+0.03±0.004	+0.018±0.003	+0.047±0.006	+0.047±0.006

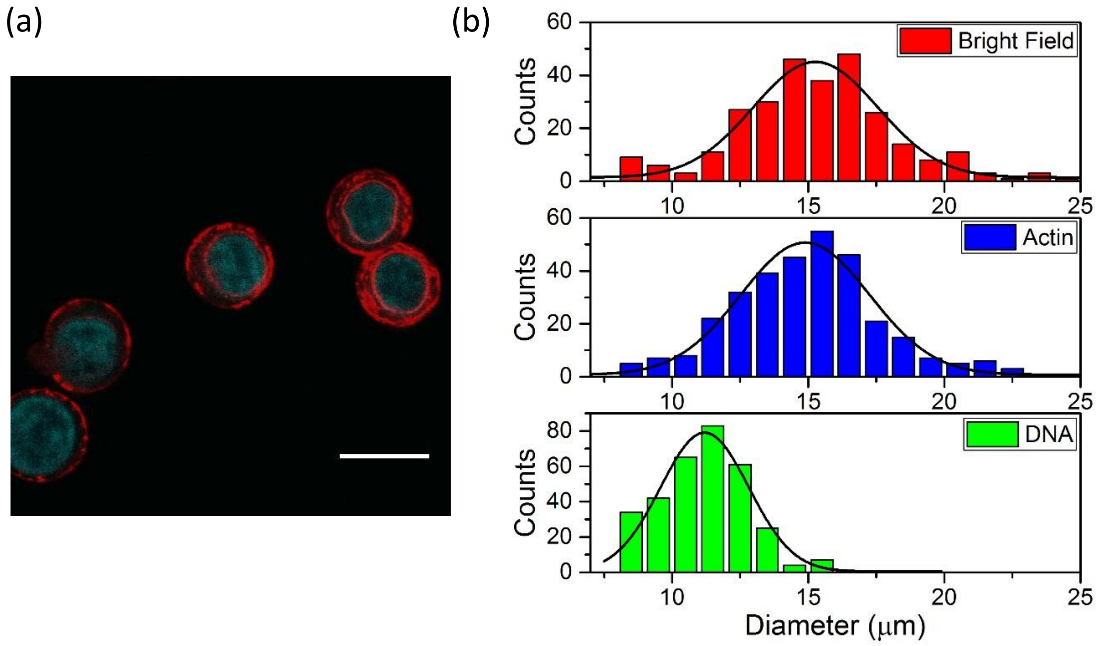

Figure S3: *(a) Confocal fluorescence image of SW480 cells, stained for actin (red) and DNA (blue). (b) Histograms showing the cell diameter found using the bright field image, actin cortex diameter from the actin stain, and the nucleus diameter from the DNA staining.*

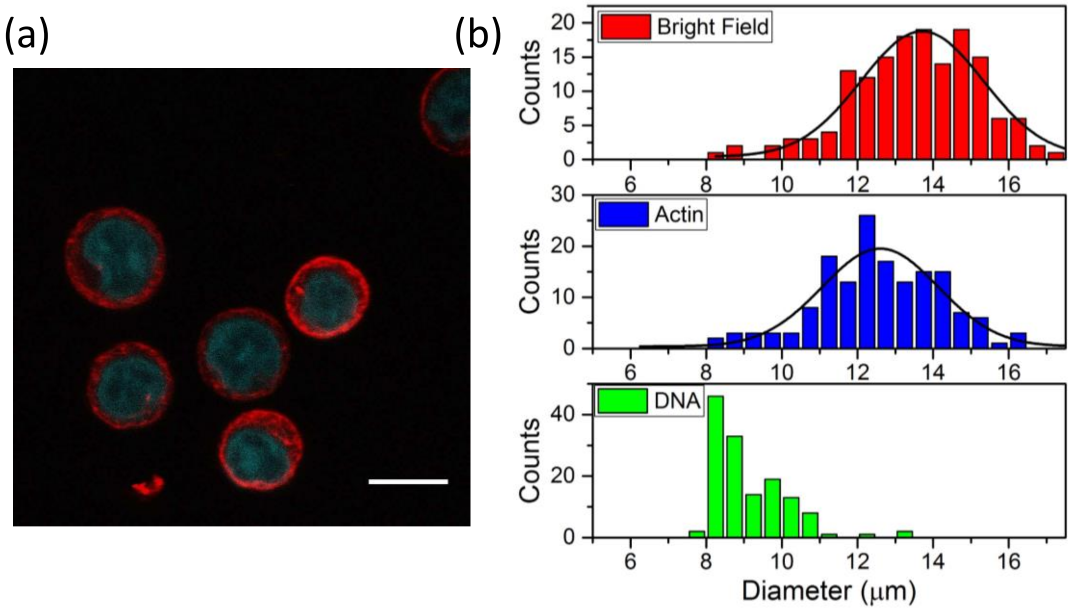

Figure S4: *(a) Confocal fluorescence image of SW620 cells, stained for actin (red) and DNA (blue). (b) Histograms showing the cell diameter found using the bright field image, actin cortex diameter from the actin stain, and the nucleus diameter from the DNA staining.*

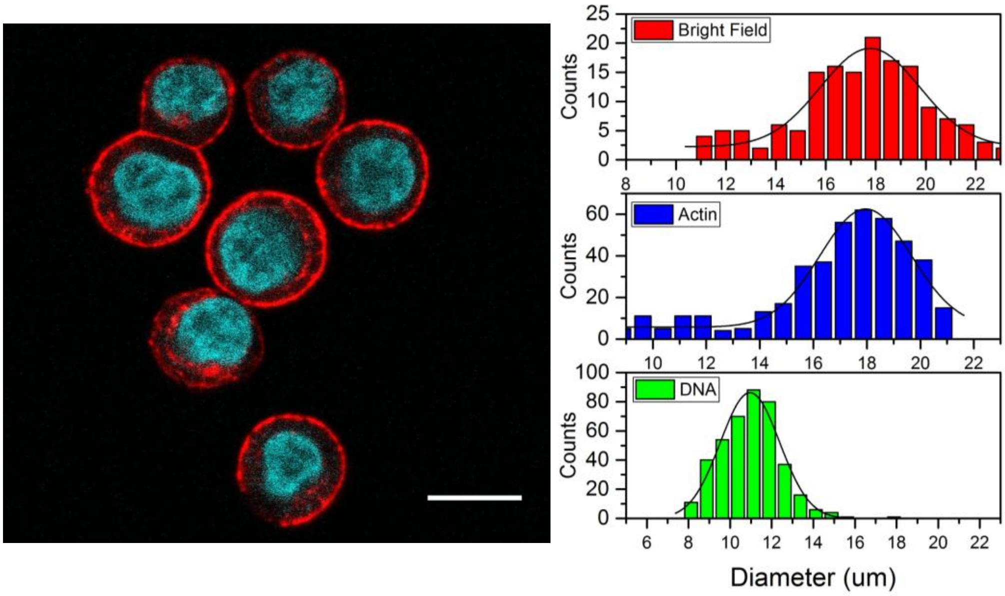

Figure S5: *(a) Confocal fluorescence image of HT29 cells, stained for actin (red) and DNA (blue). (b) Histograms showing the cell diameter found using the bright field image, actin cortex diameter from the actin stain, and the nucleus diameter from the DNA staining.*

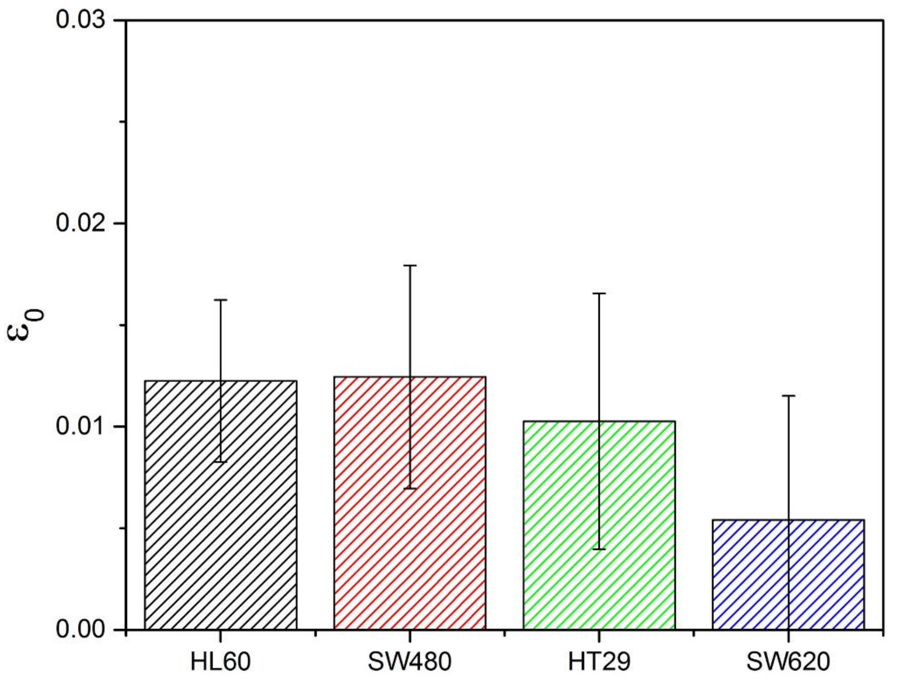

Figure S6: *(a) The average initial strain magnitude ε_0 of HL60, SW480, SW620 and HT29 cell populations before they were deformed at the SP. The error bars denote the standard error SE, statistical t-tests were done to determine the level of significance. Showing the initial strain of the cells ε_0. Each cell comparison has no significance (ns) (p>0.05).*

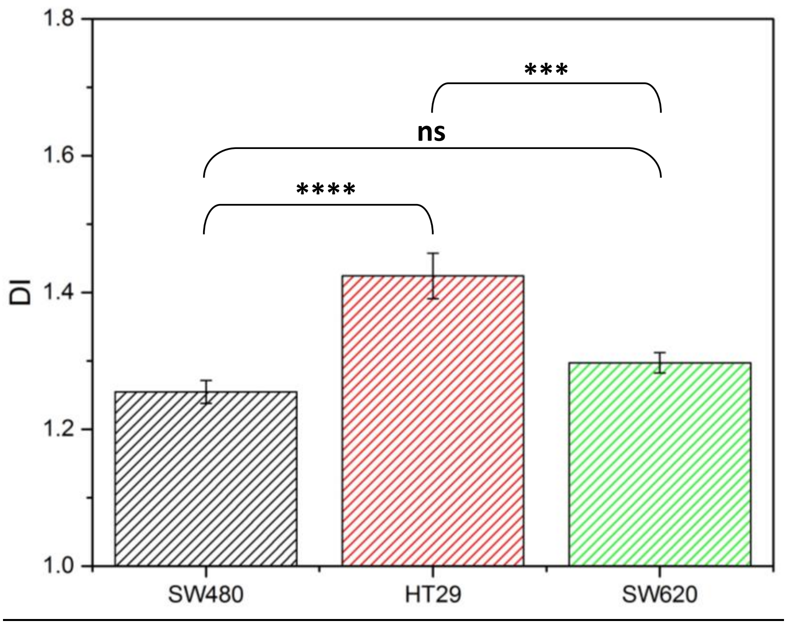

Figure S7: *(a) The average DI of SW480, HT29 and SW620 cells deformed at 5 µl/min in a shear-dominant regime (~33 cP). The error bars denote the standard error SE, statistical t-tests were done to determine the level of significance. HT29 shows extremely significant difference to SW480 ($p = 7.5 \cdot 10^{-6}$) and SW620 ($p = 5.5 \cdot 10^{-4}$), whereas any difference between SW480 and SW620 is not significant (p=0.058).*

Table S2: *Summary of p-values comparing of HL60, SW480, SW620 and HT29 cell populations. Statistical t-tests were done to determine if the level of significance of differences between different parameters which includes: initial cell diameter A, the maximum strain ε_{max}, the final strain ε_{∞} and the relaxation time τ_r were extracted from deformation traces of single cells deforming at 5 µl/min in a shear dominant regime (~33 cP).*

		A (µm)		ε_{∞}		ε_{max}		τ_r		ε_0	
		**p-value**	**Sig.**	**p-value**	**Sig.**	**p-value**	**Sig.**	**p-value**	**Sig.**	**p-value**	**Sig.**
**HL60**	**SW480**	2.77E-13	****	0.01074	*	4.23E-17	****	7.03E-18	****	9.78E-01	ns
**HL60**	**HT29**	5.09E-12	****	1.44E-02	*	3.78E-09	****	2.09E-19	****	0.33807	ns
**HL60**	**SW620**	0.0022	**	2.35E-02	*	2.10E-11	****	7.38E-10	****	0.35094	ns
**SW480**	**HT29**	0.07687	*	1.44E-05	****	7.03E-04	***	5.93E-01	ns	0.38193	ns
**SW480**	**SW620**	1.35E-20	****	3.66E-05	****	1.90E-02	*	8.92E-01	ns	3.94E-01	ns
**SW620**	**HT29**	1.43E-22	****	9.05E-01	ns	2.80E-01	ns	5.19E-01	ns	9.84E-01	ns

Table S3: *Summary of the average nuclear diameter of populations of HL60, SW480, HT29 and SW620 cells. Measured using confocal images, examples shown in Figures S4-6. The nuclear ratio of each cell line was also measured ($A_{nucleus}/A_{cell}$).*

	Nuclear Diameter (µm)	Nuclear Ratio
HL60	8.9±0.1	0.55±0.02
SW480	11.2±0.1	0.72±0.01
HT29	11.0±0.1	0.63±0.01
SW620	9.2±0.1	0.67±0.06
